# Human African trypanosomiasis control: Achievements and challenges

**DOI:** 10.1371/journal.pntd.0005454

**Published:** 2017-04-20

**Authors:** Serap Aksoy, Phillipe Buscher, Mike Lehane, Philippe Solano, Jan Van Den Abbeele

**Affiliations:** 1 Department of Epidemiology of Microbial Diseases, School of Public Health, Yale University, New Haven, Connecticut, United States of America; 2 Department of Biomedical Sciences, Institute of Tropical Medicine, Antwerp, Belgium; 3 Department of Vector Biology, Liverpool School of Tropical Medicine, Liverpool, United Kingdom; 4 Institut de Recherche pour le Développement (IRD), Montpellier, France; Baylor College of Medicine, Texas Children's Hospital, UNITED STATES

## Abstract

Sleeping sickness, also known as human African trypanosomiasis (HAT), is a neglected disease that impacts 70 million people living in 1.55 million km^2^ in sub-Saharan Africa. Since the beginning of the 20th century, there have been multiple HAT epidemics in sub-Saharan Africa, with the most recent epidemic in the 1990s resulting in about half a million HAT cases reported between 1990 and 2015. Here we review the status of HAT disease at the current time and the toolbox available for its control. We also highlight future opportunities under development towards novel or improved interventions.

Human African trypanosomiasis (HAT) is a neglected disease that impacts 70 million people living in 1.55 million km^2^ of sub-Saharan Africa [1]. Since the beginning of the 20th century, there have been three major HAT epidemics, the most recent in the 1990s resulting in about 500,000 HAT cases reported between 1990 and present [2,3]. The disease is caused by two distinct subspecies of the African trypanosomes transmitted by tsetse flies (*Glossina* spp.: Diptera). In east and southern Africa, *Trypanosoma brucei rhodesiense* causes the acute Rhodesiense form of the disease, while in central and west Africa. *T*. *b*. *gambiense* causes the chronic Gambiense form of the disease (about 95% of all reported HAT cases). The disease normally affects remote rural communities where people get exposed to the bite of the tsetse during their daily outdoor activities. Unless treated, HAT disease is normally fatal. Besides HAT, related parasites, such as *T*. *b*. *brucei*, *T*. *congolense*, *T*. *vivax*, *T*. *evansi*, *and T*. *equiperdum*, cause wasting diseases in livestock, termed animal African trypanosomiasis (AAT), which result in major economic losses in the concerned countries [4].

An ambitious campaign led by WHO, many nongovernmental organizations (NGOs), and a public–private partnership with Sanofi-Aventis and Bayer that donated the necessary drugs for distribution in affected countries reduced the global incidence of Gambiense HAT to <3,000 cases in 2015. Based on the success of the control campaign, there are now plans to eliminate Gambiense HAT as a public health problem by 2020 [5]. Gambiense HAT is generally considered to be an anthroponosis, and hence control has relied heavily on active and/or passive case detection and treatment programs [6]. Control of Rhodesiense HAT has been more complex, as disease transmission involves domestic animals, which serve as reservoirs for parasite transmission by the tsetse vector. Hence, for Rhodesiense HAT, control of the parasite in the domestic reservoirs, and/or reduction of tsetse vector populations, plays a key part, with medical interventions being used only for humanitarian purposes.

Despite extensive research into the biology of the trypanosome, the toolbox for diagnostics and treatment of sleeping sickness had remained extremely small and plagued with difficulties [7]. However, the recent epidemic has mobilized financial resources available for basic and applied research, which has led to new knowledge on both parasite and tsetse vector physiology, genetics, and genomics and expanded the prospects for translational science for sustainable HAT control. Below, we review some of the progress made in the last decade towards growing the toolbox for HAT elimination.

## Controlling disease in the mammalian host

Achieving disease control in the mammalian host has been difficult given the lack of mammalian vaccines due to a process of antigenic variation the parasite displays in its mammalian host. Hence, accurate diagnosis of the parasite and staging of the disease are important, particularly because of the toxicity of current drugs. Although powerful molecular diagnostics have been developed in research settings, few have yet reached the patient or national control programs [8]. For Gambiense HAT, a sensitive and specific test is available for active serological screening by mobile teams [9]. However, when prevalence becomes low, targeted door-to-door surveys may become an attractive alternative to mass screening [10]. It wil also become crucial to integrate passive case detection in fixed health centers [5]. The development of individual rapid sero-diagnostic tests (RDTs) will certainly facilitate the involvement of fixed health centers in passive case detection, yet their diagnostic accuracy remains uncertain [11]. Screening for *T*. *b*. *rhodesiense* infection still relies on clinical features in the absence of serological tests available for field use. Although recent improvements have been made to parasitological confirmatory tests, their sensitivity remains insufficient in Gambiense HAT [12]. Regarding treatment, the introduction of the eflornithine-nifurtimox combination therapy (NECT) has made treatment of Gambiense HAT safer and more efficacious [13].

## Reducing vector populations

While vector control is essential for Rhodesiense HAT, it has not played a major role in Gambiense HAT, as it was considered too expensive and difficult to deploy in the resource-poor settings of HAT foci. However, modelling, historical investigations, and practical interventions demonstrate the significant role that vector control can play in the control of Gambiense HAT [14, 6, 15], especially given the possibility of long-term carriage of trypanosomes in both human and animal reservoirs [16,17]. Recent models suggest vector control will be essential if we are to reach the set target of elimination of the disease as a public health problem by 2020 [18]. Vector control can be particularly effective at times of low endemicity during which active surveillance campaigns may be too costly to operate, and it is, so far, the only prophylactic measure existing to protect people against the infectious bite of the tsetse vector. In addition, the use of commercially-available loop-mediated isothermal amplification (LAMP) kits as a highly sensitive tool for xenomonitoring has also been suggested to identify potential sleeping sickness transmission sites, especially during periods of low endemicity [19].

For vector reduction efforts, the use of Sterile Insect Technique continent-wide has been suggested by the African Union, following the success of the eradication program in Zanzibar [20]. However, the feasibility of the application of this method continent-wide has come into question given the diversity of species that can transmit the parasite, its high cost, and dependence on major infrastructure such as large insectaries, irradiation facilities, and airplanes [21]. It has been possible to modify the existing insecticide-treated targets to produce a more cost-efficient and sustainable vector control method for use in HAT foci. Tiny targets consisting of a small square of blue cloth flanked by a similar-sized piece of black netting have been shown to be efficacious and more cost-effective than traps or large targets typically used in control campaigns for the Gambiense vector species [14, 22]. To monitor the efficacy and sustained impact of tsetse control interventions, sensitive serological tools are being developed using tsetse-saliva–based biomarkers [11, 23]. A major advancement that has fueled research into the fundamental aspects of tsetse and trypanosome transmission biology has been the completion of the genome sequence of *G*. *morsitans morsitans* [24] and five additional vector species [41]. This information has been mined to understand the olfactory [25, 26], symbiotic [27], and reproductive [28] physiologies of tsetse, with the goal of identifying targets suitable for population reduction efforts. Knowledge on olfactory physiology can be harnessed to enhance the efficacy of traps and targets by identifying novel attractants, while the obligate dependence of tsetse on their endosymbionts provides a weak link in this vector’s ability to reproduce, both providing ideal targets for new vector control methods.

In addition, there has been growing knowledge on the population genetics of tsetse vectors in disease-endemic areas in Africa. This new information can help identify natural barriers to fly dispersal and routes and hence can provide important information for field control programs that aim to reduce vector densities through traditional tools, such as traps/targets, Sterile Insect Technique, or aerial sprays [29, 30].

One fundamental reason vector control methods are highly effective in tsetse is the already low reproductive capacity this insect has due to its viviparous reproductive physiology. The molecular and biochemical aspects of tsetse’s viviparity and its dependence on the obligate symbiont *Wigglesworthia*-provided products are being unraveled. This fundamental knowledge provides many new targets for interference with tsetse fecundity to reduce tsetse populations [28].

## Controlling the parasite in the tsetse vector

The increased knowledge of tsetse and trypanosome genomics has also expanded knowledge on the molecular aspects of host–parasite interactions with several practical implications. It has been shown that an important bottleneck for parasite transmission occurs early in the infection process in the tsetse’s midgut, characterized by a tug-of-war between trypanosome immune modulatory activities and the tsetse’s antiparasitic immune responses [31, 32]. The molecular details of the complex host–parasite interactions that eventually enable the trypanosomes to colonize the flies [33, 34], followed by modulation of the fly saliva immunomodulatory components by parasites, and the impact of this modification on trypanosome transmission dynamics in the host bite site favoring parasite transmission are all being unraveled and provide novel points of interference for the parasite’s transmission [35, 23]. One approach whereby the parasite infection can be eliminated from tsetse populations involves the ability to engineer refractoriness in tsetse [36, 37]. Towards that end, one commensal symbiont of tsetse present in the midgut, *Sodalis*, has been identified, and a genetic modification system has been developed [38]. Candidate antitrypanosomal molecules have also been identified and expressed in *Sodalis* to reduce trypanosome infections as a novel approach [39, 40]. Many of these early-stage fundamental discoveries have been achieved as a consequence of the applications of–omics technologies to the field of tsetse and trypanosomes and have opened up the feasibility of novel targets for new innovative approaches.

## Highlights

1. The Gambiense HAT epidemics that ravaged sub-Saharan Africa in the 1990s have been controlled, leading the way to an elimination phase. The elimination of Rhodesiense HAT poses challenges because of the vast animal reservoirs.
2. Simplification, standardization, and proper test evaluation of diagnostic tools in the target setting should be an important focus for future development to maintain low endemicity and to monitor disease prevalence in the post elimination phase.
3. A safe and oral drug that cures both disease stages and both disease forms is needed.
4. Control strategies will progressively shift from active case detection by mobile teams towards passive case detection by fixed health centers.
5. The capacity built towards HAT diagnosis and treatment should be preserved at times of low endemicity and post elimination.
6. Application of vector control alongside medical interventions will be needed to achieve the targets within the set time frame.
7. The molecular targets discovered through parasite and tsetse genomics/genetics studies form the pipeline for new drugs, potential antitsetse-based vaccines, and fly inhibitory compounds, which should be explored as novel biological control methods.
8. The time has come for donors, private companies, and all stakeholders to commit to the elimination of HAT, in order to avoid resurgence as seen in the past.
